# Genetic analysis of protein content and oil content in soybean by genome-wide association study

**DOI:** 10.3389/fpls.2023.1182771

**Published:** 2023-06-06

**Authors:** Hui Jin, Xue Yang, Haibin Zhao, Xizhang Song, Yordan Dimitrov Tsvetkov, YuE Wu, Qiang Gao, Rui Zhang, Jumei Zhang

**Affiliations:** ^1^ Institute of Forage and Grassland Sciences, Heilongjiang Academy of Agricultural Sciences, Harbin, China; ^2^ Horticultural Branch of Heilongjiang Academy of Agricultural Sciences, Harbin, China

**Keywords:** genome-wide association study (GWAS), marker-assisted selection (MAS), protein content, oil content, soybean, *Glycine max L.*

## Abstract

Soybean seed protein content (PC) and oil content (OC) have important economic value. Detecting the loci/gene related to PC and OC is important for the marker-assisted selection (MAS) breeding of soybean. To detect the stable and new loci for PC and OC, a total of 320 soybean accessions collected from the major soybean-growing countries were used to conduct a genome-wide association study (GWAS) by resequencing. The PC ranged from 37.8% to 46.5% with an average of 41.1% and the OC ranged from 16.7% to 22.6% with an average of 21.0%. In total, 23 and 29 loci were identified, explaining 3.4%–15.4% and 5.1%–16.3% of the phenotypic variations for PC and OC, respectively. Of these, eight and five loci for PC and OC, respectively, overlapped previously reported loci and the other 15 and 24 loci were newly identified. In addition, nine candidate genes were identified, which are known to be involved in protein and oil biosynthesis/metabolism, including lipid transport and metabolism, signal transduction, and plant development pathway. These results uncover the genetic basis of soybean protein and oil biosynthesis and could be used to accelerate the progress in enhancing soybean PC and OC.

## Background

Soybean (*Glycine max* L.) is an important economic crop in the world and is a major source of vegetable oil and feed protein (Liu et al., 2008). Dry soybean seeds are composed of approximately 40% protein content (PC) and 20% oil content (OC). Increasing seeds’ PC (Li et al., 2021) and OC (Zhang et al., 2019) is an important breeding objective for soybean. The improvement of PC and OC is challenging due to their polygenic inheritance (Li et al., 2020). China, Russia, the United States, and Canada are the major soybean-producing countries (Xue et al., 2022). Although soybean production has been improved largely by traditional breeding, it is still not enough to meet the demand (Li et al., 2019; Li et al., 2020).

PC and OC are typical quantitative traits and are controlled by two major loci on chromosomes 15 and 20 and other genes with minor effects (Wang et al., 2020), and they are influenced by both environmental and genetic factors (Clemente and Cahoon, 2009; Pathan et al., 2013). Marker-assisted selection (MAS) is an effective method for the genetic improvement of PC and OC (Jiang et al., 2018; Karikari et al., 2019). The reliability and efficacy of MAS depend on the number and phenotypic effects of the genes/quantitative trait loci (QTL) on the corresponding trait (Zhang et al., 2018; Karikari et al., 2019; Wang et al., 2020). Thus, QTL mapping for soybean seed PC and OC plays a vital role in soybean MAS breeding (Zafar et al., 2019; Wang et al., 2020). Over 200 and 300 QTLs for PC and OC have been deposited in SoyBase (http://www.soybase.org) (Brown et al., 2021), and over 30 genes and functional markers for PC and OC have been cloned and developed, such as *GmbZIP123*, *GmMYB73*, *GmDOF4*, *GmDOF11*, and *GmNFYA* (Li et al., 2019; Wang et al., 2020). Overexpression of *GmSDP1-4* and *GmPDAT* increased seed oil (Liu et al., 2020; Liu et al., 2020b). GA20 oxidase (*GA20OX*) and nuclear transcription factor Y subunit alpha (*NFYA*) are two key drivers of seed traits and enhanced seed size and weight and OC, respectively. *GmOLEO1* and *GmWRI1a* also significantly increased total OC and fatty acid content (Zhang et al., 2019; Zhang et al., 2022). Furthermore, several transcription factors were also found to be associated with soybean seed oil. Overexpression of *GmbZIP123*, *GmMYB73*, *GmZF351*, *GmZF392*, and *GmWRI1b* enhanced lipid content (Guo et al., 2020). Compared with genes associated with OC, fewer genes controlling PC or amino acids have been identified. These include *MGL* (a putative methionine γ-lyase), *OASS* (the cytosolic isoform of O-acetylserine sulfhydrylase), Rab5a (a small GTPase-encoding gene), and SWEETs (sugar will eventually be exported transporters) (*GmSWEET15*, *GmSWEET10a*, and *GmSWEET10b*), which played important roles in soybean seed quality through their effects on both OC and PC (Wang and Tian, 2015; Zhang et al., 2022).

Most of these QTLs were mapped by linkage mapping based on bi-parental populations, including the recombinant inbred line (RIL), F_2_, and backcross populations. However, linkage mapping was limited by the small phenotypic variation of bi-parental populations. Furthermore, traditional molecular markers used in these studies reduced the accuracy of QTL mapping due to their low densities (Rasheed et al., 2016; Liu et al., 2017; Klepadlo et al., 2019; Samanfar et al., 2019; Wang et al., 2020; He et al., 2021; Ullah et al., 2021).

Single nucleotide polymorphisms (SNPs) are more abundant with higher coverage and are markers of choice for gene discovery and MAS (Yuan et al., 2014; Rasheed et al., 2016). The development of SNP chips and next-generation sequencing (NGS) made the use of SNP quite affordable and feasible in molecular breeding. Linkage mapping and association analysis identifying genes/QTLs for complex traits are most widely used for gene discovery (Kim et al., 2010; Chan et al., 2012; Liu et al., 2016; Liu et al., 2017; Song et al., 2017; Wang et al., 2020). Currently, soybean SNP arrays are widely used in genetic analysis for yield, processing quality, and disease resistance-related traits (Akond et al., 2014; Chaudhary et al., 2015; Zhang et al., 2018; Beyer et al., 2019; Li et al., 2019; Zhang et al., 2019; Tian et al., 2020; Wang et al., 2020). In contrast to traditional bi-parental mapping, genome-wide association studies (GWASs) use natural diversity (such as wild types, landraces, and cultivars) and offer an effective and reliable way to uncover the genetic basis of complex traits (Zhu et al., 2008; Sela et al., 2014; Liu et al., 2017; Schläppi et al., 2017; Shi et al., 2017; Wang et al., 2019; Alqudah et al., 2020). GWAS has been widely used in the genetic analysis of yield, disease resistance, and quality-related traits in soybean (Vuong et al., 2015; Li et al., 2019; Wang et al., 2020; Zahid et al., 2022).

In this study, 320 soybean accessions collected mainly from the major soybean-growing countries were used to (1) identify loci underpinning PC and OC in soybean and (2) identify candidate genes for further study.

## Materials and methods

### Plant materials and field trials

A total of 320 soybean accessions from the main soybean-growing countries (including the United States, Algeria, Canada, China, Czechia, France, Germany, Hungary, Japan, Moldova, Romania, Russia, Serbia, Sweden, and Ukraine) were collected and used for the evaluation of PC and OC (**Table S1**). All 320 soybean accessions were planted at the Heilongjiang Academy of Agricultural Sciences experimental station in Harbin, China, with three replications in 2017, 2018, 2019, and 2021; Mudanjiang in 2018 and Qingan in 2018. A completely randomized block design with three replicates was used, with each line 3.0 m in length and 0.65 m apart, and with 6 cm spacing between two plants. Field management was in accordance with local field cultivation conditions.

### Phenotyping and statistical analysis for protein and oil contents

Soybean seeds were harvested from 10 plants from each genotype and subsequently used for the PC and OC determination. The Infratec 1241 NIR Grain Analyzer (FOSS, Sweden) was used to analyze three seed samples from each genotype in each replicate (20–25 g). The phenotypic values given for each accession used in this study were all the mean values of the three replicates. The best linear unbiased estimation (BLUE) for PC and OC among all environments was calculated using the R package “sommer”. The maximum, minimum, and standard deviation of the PC and OC were calculated. Analysis of variance (ANOVA) was used to compare the phenotypic values of PC and OC in each environment or jointly in multiple environments. The heritability was estimated using the entry-mean basis formula $h^{2}=\frac{\sigma_{g}^{2}}{\sigma_{g}^{2}+\frac{\sigma_{v}^{2}}{n_{e}}+\frac{\sigma_{\epsilon }^{2}}{n_{e}\times n_{r}}}$ , where $\sigma_{g}^{2}$ , $\sigma_{v}^{2}$ , and $\sigma_{\epsilon }^{2}$ is the genetic effect, environmental effect, and residual, respectively, and $n_{e}$ and $n_{e}$ is the number of environments and the number of replicates, respectively.

## Genotyping and population structure

### SNP genotyping

For QTL-seq, the genomic DNA from the seedling leaf was isolated. The DNA was genotyped by re-sequencing using the Illumina HiSeq 2500 platform (Illumina, Inc., San Diego, CA, United States) by Biomarker Biotechnology Co., Ltd. The paired-end read data (PE150) with a sequencing depth of approximately 10× of the soybean genome were generated. In total, 3814.10 giga base pairs (Gbp) of clean data, with a base call accuracy of about 93.10%, were obtained. The average comparison rate between the sample and reference genome was 98.47%, with an average coverage depth of 10× and genome coverage of 97.18%. The SNPs were filtered by minor allele frequency (MAF)< 0.05 and missing rate > 10%. Population structure was analyzed using Structure v2.3.4 (Pritchard et al., 2000). Principal component analysis (PCA) was performed and mega trees were created using Tassel v5.0 (Breseghello and Sorrells, 2006). Linkage disequilibrium (LD) decay was calculated using the full matrix and sliding window options in Tassel v5.0.

### Genome-wide association study and candidate gene identification

A mixed linear model (MLM, PCA (fixed-effect factor) + K (random-effect factor)) in Tassel v5.0 (Breseghello and Sorrells, 2006) was used to avoid spurious marker–trait associations (MTAs) as follows: *y*=*μ*+*xβ*+*u*+*e* (*y*: phenotype; *µ*: mean value; *x*: genotype; *β*: effect of the SNP; *u*: the random effects). In this study, the Bonferroni–Holm correction for multiple testing (alpha = 0.05) was too conservative, and no significant MTAs were detected. Thus, markers with the threshold for the significant associations is –log_10_ (*p*-value) ≥ 6.0. Manhattan and quantile–quantile (Q–Q) plots were drawn using CMplot. Candidate genes for PC and OC consistently identified in two or more populations were identified in this study. The following steps were conducted to identify the candidate genes. Firstly, all genes located in the LD block region around the peak SNP ( ± 480 kb based on previous LD decay analysis) of each important QTL were retrieved. Then, all available SNPs located inside these genes were searched against GenBank using the flanking sequences of the SNPs (including the LD decay interval) significantly associated with PC and OC. The genes (except the hypothetical protein, transposon protein, and retrotransposon protein) that were identified with MTAs of non-synonymous SNPs in the coding region that could further lead to sense mutations were considered. Thus, genes involved in protein and oil biosynthesis/metabolism were regarded as high-confidence candidate genes for PC and OC.

Quantitative real-time PCR (qRT-PCR) was performed to test the expression of selected candidate genes in the accessions with extreme PC and OC. All seeds were sampled for RNA extraction after maturity. cDNA was synthesized using the HiScript II 1st Strand cDNA synthesis kit and the primers were designed using Primer 5.0 software. PCR was conducted in a volume of 20 µL (2 µL cDNA, 10 µL ChamQ Universal SYBR qPCR Master Mix, and 0.4 µL of each primer (µM)) (**Table S2**). All assays were conducted in two independent experiments with three repetitions.

## Results

### Phenotypic evaluation

OC and PC showed continuous and significant variations in the 320 soybean accessions. The BLUE values of OC and PC were 21.0% (from 16.7% to 22.6%) and 41.1% (from 37.8% to 46.5%), respectively (**Figure S1**; **Table S1**). The standard deviation and coefficient of variation of OC and PC were 0.86% (0.041) and 1.38% (0.034) across all environments, respectively. The OC was negatively correlated with PC (–0.532, *p*<0.001). ANOVA indicated highly significant effects (*p*<0.01) of genotypes, environments, and genotype × environment interactions on PC and OC (**Table 1**). The SNP-based heritability for OC and PC was 0.77 and 0.78, respectively.

**Table 1:** ANOVA analysis for the protein content and oil content in 320 soybean accessions.

Source of variation	df	F-value	
		PC	OC
Genotypes	319	120.4**	24.4**
Environments	5	380.9**	98.5**
Replicates (nested in environments)	2	18.2**	5.3**
Genotypes*Environments	1594	9.2**	4.3**
Error	1425	–	–

* and ** indicate significance at 0.05 and 0.01 levels.

### Genotyping, population structure, and linkage disequilibrium decay analysis

**Figure 1:**
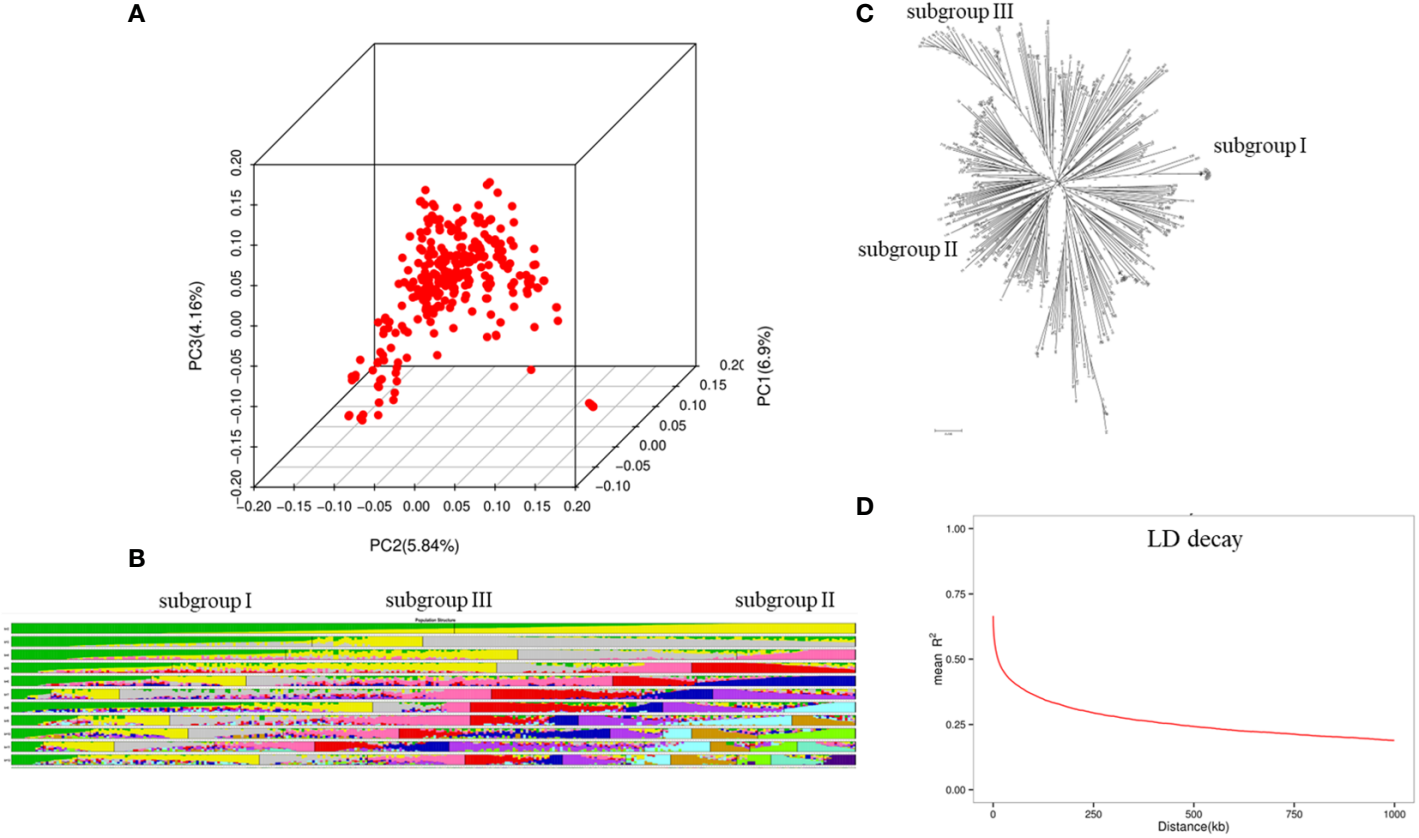
Population structure analysis for the 320 soybean accessions. **(A)** principal component analysis (PCA) plots; **(B)** population structure analysis from k=2 to 12; **(C)** neighbor-joining (NJ) tree; **(D)** LD decay.

In total, 3,290,923 polymorphic SNPs after filtration (MAF< 0.05, missing rate > 0.1) were used for GWAS. Chromosome 18 had the highest number of SNPs (233,764), whereas chromosome 11 had the lowest number of SNPs (74,209). The average marker density was 304.0 marker/kb on the genome-wide scale. The population structure divided the 320 soybean accessions into three subgroups, namely subgroup I, II, and III. Of these, subgroup I consisted of 156 accessions from China, Russia, and Ukraine, subgroup II had 102 accessions from the United States and Canada, and subgroup III comprised 62 accessions from Germany, France, and Czechia (**Figure 1**). The neighbor-joining (NJ) tree results also suggested that the 320 soybean accessions could be divided into three subgroups. PCA analysis indicated that the top three PCAs explained 22.1%, 18.3%, and 12.5% of the total variances. In addition, PCA results indicated that all 320 soybean accessions could belong to the three subgroups. The NJ tree and PCA analysis validated the results of the population structure analysis. The average LD decay of the genome was about 480 kb according to the locally estimated scatterplot smoothing (LOESS) curve (**Figure 1**).

### Genome-wide association study

In total, 29 loci for OC were identified on all chromosomes except for chromosome 9, and each explained 5.1%–16.3% of the total phenotypic variances. For OC, chromosome 20 contained three loci, and chromosomes 2, 6, 7, 11, 12, and 19 contained two loci, whereas chromosomes 3, 4, 5, 8, and 10 each had only one locus. For PC, chromosome 11 contained four loci, and chromosomes 2, 5, and 20 contained two loci, whereas chromosomes 1, 3, 4, 6, 7, 8, 9, 13, 14, 15, 16, and 18 each had only one locus. Twenty-three loci for PC were identified on all chromosomes except for chromosomes 12, 17, and 19, and explained 3.4%–15.4% of the phenotypic variances. Of these, six pleiotropic loci including *qOC2.2* (44.7–49.2 Mb) and *qPC2.2* (45.0–50.8 Mb); *qOC4.1* (41.3–45.3 Mb) and *qPC4.1* (44.6–5.0.7 Mb); *qPC5.2* (44.1–48.7 Mb) and *qOC5.1* (46.8–47.7 Mb); *qOC13.1* (19.9–25.0 Mb) and *qPC13.1* (24.3–28.6 Mb); *qOC14.1* (43.5–49.1 Mb) and *qPC14.1* (47.2–49.8 Mb); and *qOC20.3* (43.9–49.0 Mb) and *qPC20.2* (45.0–48.0 Mb) were significantly associated with both PC and OC.

**Figure 2:**
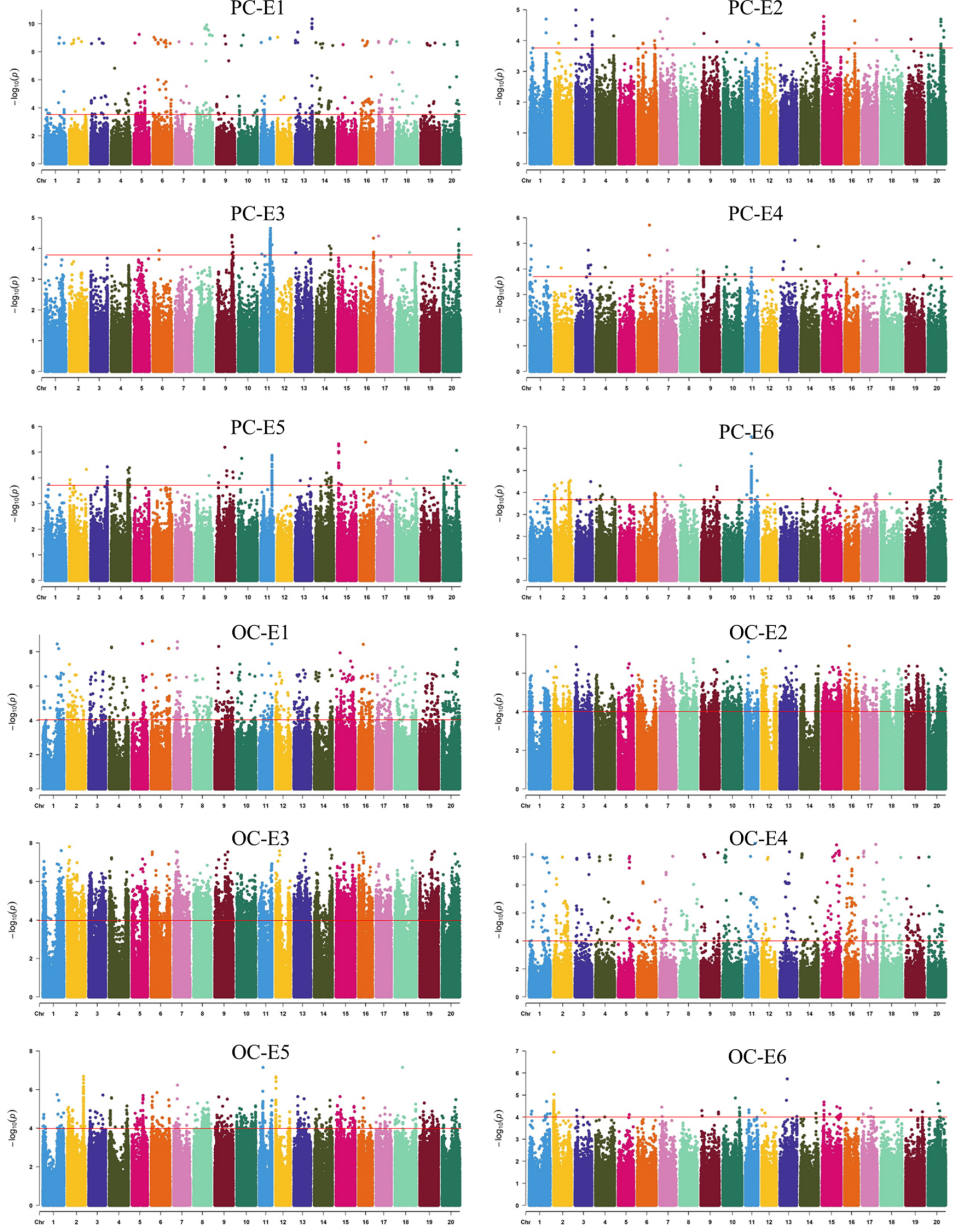
The distribution of protein content and oil content across all environments. PC: protein content; OC: oil content; E1, E2, E3 E4, E5, and E6 indicate 2017 Harbin, 2018 Harbin, 2019 Harbin, 2021 Harbin, 2018 Mudanjiang, and 2018 Qingan, respectively.

For PC, *qPC5.2*, *qPC9.1*, *qPC10.1*, *qPC11.1*, *qPC11.2*, *qPC11.3*, *qPC11.4*, *qPC14.1*, and *qPC16.1* were identified across all six environments, *qPC1.1, qPC3.1, qPC4.1, qPC18.1, qPC20.1*, and *qPC20.2* were identified in five environments, and the other loci were identified in three or four environments. For OC, *qOC1.2*, *qOC6.2, qOC11.1, qOC12.1, qOC13.1, qOC15.1, qOC16.1*, and *qOC20.1* were identified in five environments, whereas *qOC1.1, qOC2.2*, and *qOC3.1* were identified in only one environment. The other loci were identified in two or three environments (**Tables 2**, **3**, **S2**; **Figures 2**, **S2**).

**Table 2:** Loci for protein content in 320 soybean accessions by association analysis.

Loci	Chromosome	Interval		Environment	*p*-value		R^2^		Favorable allele	Effect	Reference
		Start (Mb)	End (Mb)		Lower	Higher	Lower	Higher			
*qPC1.1*	1	43.2	48.7	E1, E2, E3, E5, E6	7.7E-04	2.6E-09	4.9%	13.5%	G	3.5–10.5	
*qPC2.1*	2	0.3	2.9	E1, E5, E6	9.9E-04	1.4E-09	4.5%	13.7%	G	2.3–9.6	
*qPC2.2*	2	45.0	50.8	E1, E2, E3, E6	9.2E-04	2.9E-05	4.5%	6.9%	G	3.6–12.8	Li et al. (2019)
*qPC3.1*	3	47.3	50.4	E1, E2, E3, E5, E6	9.8E-04	1.6E-05	4.4%	6.0%	G	–6.3–3.2	
*qPC4.1*	4	44.6	50.7	E1, E2, E4, E5, E6	8.7E-04	6.1E-05	4.7%	6.3%	G	3.0–6.5	
*qPC5.1*	5	11.0	14.9	E1, E3, E4, E6	9.2E-04	3.1E-06	4.6%	8.8%	A	3.1–7.8	
*qPC5.2*	5	44.1	48.7	E1, E2, E3, E4, E5, E6	9.9E-04	3.9E-05	4.5%	6.6%	G	–8.6–2.3	
*qPC6.1*	6	45.1	47.4	E1, E2, E5, E6	8.2E-04	1.0E-06	4.6%	9.2%	A	2.5–6.5	
*qPC7.1*	7	3.9	8.4	E6, E5, E4, E3	9.9E-04	2.7E-09	3.4%	13.5%	A	2.1–7.6	Pathan et al. (2013); Zhang et al. (2021)
*qPC8.1*	8	9.9	11.8	E1, E3, E5, E6	7.7E-04	1.8E-10	4.8%	15.4%	G	1.6–7.2	
*qPC9.1*	9	6.8	9.8	E1, E2, E3, E4, E5, E6	9.7E-04	1.7E-05	4.6%	7.3%	G	–6.5–1.2	
*qPC10.1*	10	6.0	10.8	E1, E2, E3, E4, E5, E6	8.6E-04	3.5E-09	4.5%	13.3%	G	3.2–6.4	
*qPC11.1*	11	0.6	3.9	E1, E2, E3, E4, E5, E6	9.8E-04	1.7E-06	4.5%	9.3%	G	2.3–5.8	
*qPC11.2*	11	34.3	37.4	E1, E2, E3, E4, E5, E6	9.4E-04	1.6E-05	3.5%	6.1%	G	2.0–7.6	
*qPC11.3*	11	42.0	45.5	E1, E2, E3, E4, E5, E6	9.8E-04	1.5E-05	4.5%	7.3%	A	2.1–8.5	
*qPC11.4*	11	47.1	51.9	E1, E2, E3, E4, E5, E6	9.9E-04	9.9E-06	3.6%	7.6%	A	–5.6–1.2	
*qPC13.1*	13	24.3	28.6	E6, E4, E3	9.3E-04	2.2E-10	3.5%	15.3%	A	–6.4–2.1	Mao et al. (2013); Zhang et al. (2021)
*qPC14.1*	14	47.2	49.8	E1, E2, E3, E4, E5, E6	9.3E-04	4.5E-05	4.5%	6.6%	A	3.4–6.4	
*qPC15.1*	15	1	4.9	E6, E5, E3	8.7E-04	1.2E-04	4.5%	5.9%	G	3.1–7.5	Pathan et al. (2013); Phansak et al. (2016); Warrington et al. (2015); Zhou et al. (2015); Zhang et al. (2021)
*qPC16.1*	16	10.5	14.7	E1, E2, E3, E4, E5, E6	9.3E-04	4.1E-06	4.6%	8.1%	A	2.5–7.6	
*qPC18.1*	18	42.1	46.8	E1, E2, E4, E5, E5	9.3E-04	2.2E-06	4.6%	8.6%	G	–9.1–2.7	
*qPC20.1*	20	34.4	40.8	E2, E3, E4, E5, E6	9.3E-04	2.0E-09	4.5%	13.5%	G	2.5–7.5	Zhang et al. (2019)
*qPC20.2*	20	45.0	48.0	E2, E3, E4, E5, E6	9.9E-04	6.1E-07	4.4%	9.7%		–6.5–3.2	

PC: protein content; E1, E2, E3, E4, E5, and E6 indicate 2017 Harbin, 2018 Harbin, 2019 Harbin, 2021 Harbin, 2018 Mudanjiang, and 2018 Qingan, respectively.

**Table 3:** Loci for oil content in 320 soybean accessions by association analysis.

Loci	Chromosome	Interval		Environment	*p*-value		R^2^		Favorable allele	Effect	Reference
		Start (Mb)	End (Mb)		Lower	Higher	Lower	Higher			
*qOC1.1*	1	10.9	13.6	E2	2.5E-08	9.9E-07	9.0%	11.8%	G	–7.6–1.2	
*qOC1.2*	1	37.0	37.9	E3, E4, E3, E2	1.1E-10	2.0E-07	10.2%	15.5%	G	3.6–9.8	
*qOC2.1*	2	31.8	33.5	E6, E3, E4	7.6E-07	5.4E-08	9.2%	11.0%	G	2.5–7.9	
*qOC2.2*	2	44.7	49.2	E2	9.5E-07	1.1E-07	9.0%	10.5%	G	3.9–11.2	Zhang et al. (2019)
*qOC3.1*	3	11.2	11.7	E2	9.8E-07	6.6E-07	9.1%	9.3%	A	3.2–9.6	
*qOC4.1*	4	41.3	45.3	E2, E4, E3	5.2E-07	1.5E-10	9.8%	15.5%	A	2.1–8.9	
*qOC5.1*	5	46.8	47.7	E4, E5	3.7E-07	3.2E-07	9.7%	9.9%	G	2.0–7.8	
*qOC6.1*	6	9.6	10.4	E4, E3, E2	4.5E-07	6.0E-09	9.6%	12.5%	A	–9.5–2.3	Zhang et al. (2019)
*qOC6.2*	6	19.6	20.1	E2, E5, E3, E4	6.1E-05	2.4E-09	6.2%	13.3%	G	3.2–5.9	Hyten et al. (2004); Diers et al. (1992); Zhang et al. (2021)
*qOC7.1*	7	11.7	15.6	E2, E3, E4	8.7E-07	8.9E-11	9.1%	15.5%	G	2.1–9.5	Li et al. (2019)
*qOC7.2*	7	28.7	30.9	E3, E4, E2	4.9E-07	3.2E-07	9.5%	10.1%	G	5.9–7.9	
*qOC8.1*	8	8.3	8.4	E6, E2, E5	5.3E-04	2.2E-05	5.1%	7.0%	A	3.1–8.8	Zhang et al. (2021); Lu et al. (2013); Pathan et al. (2013)
*qOC10.1*	10	28.5	31.4	E3, E5, E4	2.7E-07	9.2E-11	9.9%	15.5%	G	–6.9–2.3	
*qOC11.1*	11	8.5	11.0	E3, E4, E2, E6	9.1E-11	2.7E-07	10.1%	15.3%	G	3.6–8.8	
*qOC11.2*	11	26.0	29.8	E3, E2,	8.0E-08	3.9E-07	9.6%	11.0%	G	4.2–8.8	
*qOC12.1*	12	2.1	7.4	E6, E2, E3, E4	1.1E-10	7.4E-07	9.2%	15.3%	G	4.3–9.0	
*qOC12.2*	12	9.7	10.1	E5, E2	1.1E-04	3.1E-06	5.9%	8.3%	A	2.5–6.4	Zhang et al. (2021)
*qOC13.1*	13	19.9	25.0	E3, E5, E4, E2	7.8E-09	9.4E-07	9.1%	12.5%	A	5.5–9.8	
*qOC14.1*	14	43.5	49.1	E4, E3, E2	7.2E-11	9.0E-07	9.0%	15.7%	A	5.4–9.6	
*qOC15.1*	15	42.7	49.6	E3, E2, E4, E5	1.9E-10	9.7E-07	9.0%	15.2%	G	5.3–9.0	
*qOC16.1*	16	5.0	7.6	E3, E2, E4, E5	1.3E-10	9.0E-07	9.1%	15.2%	G	5.0–9.1	
*qOC16.2*	16	31.6	37.7	E2, E3	1.1E-10	5.0E-07	9.4%	15.3%	G	4.9–9.2	
*qOC17.1*	17	43.3	49.3	E2, E3, E4	9.1E-07	2.5E-10	9.0%	16.3%	A	4.9–9.9	
*qOC18.1*	18	10.8	11.1	E2, E3, E4	7.8E-07	3.1E-08	9.3%	11.7%	G	6.3–9.8	
*qOC19.1*	19	0.7	3.6	E4, E2, E5	7.0E-07	3.9E-07	9.2%	11.2%	G	–8.5–4.3	
*qOC19.2*	19	43.9	46.8	E2, E4	7.6E-07	2.8E-08	9.1%	12.9%	G	7.6–9.8	Chapman et al. (2003); Li et al. (2019)
*qOC20.1*	20	0.5	3.2	E2, E3, E5, E4	7.9E-07	9.6E-08	9.1%	11.1%	A	8.2–9.5	
*qOC20.2*	20	31.5	31.9	E2, E4	1.0E-04	5.6E-06	5.9%	7.8%	G	3.5–6.4	Bachlava et al. (2009); Zhang et al. (2021)
*qOC20.3*	20	43.9	49.0	E2, E4, E3	1.2E-08	8.7E-07	9.1%	12.1%	G	6.5–8.9	

OC: oil content; E1, E2, E3 E4, E5, and E6 indicate 2017 Harbin, 2018 Harbin, 2019 Harbin, 2021 Harbin, 2018 Mudanjiang, and 2018 Qingan, respectively.

### Candidate genes underpinning protein and oil content in soybean

In total, nine candidate genes for OC and PC were identified and showed significant differential expression between the accessions with contrasting phenotypes (**Table S2**). For *Glyma.03G261000* and *Glyma.07G137400*, the gene expression was 1.3–5.9-fold higher in accessions with higher PC and OC than in accessions with lower PC and OC. In contrast, the gene expressions were 1.2- to 5.4-fold lower in accessions with higher PC and OC than in accessions with lower PC and OC for the gene *Glyma.06G263800*, *Glyma.08G107800*, *Glyma.10G065000*, *Glyma.12G014800*, *Glyma.13G119800*, *Glyma.15G049200*, and *Glyma.20G189300* (**Tables 4**, **S3**; **Figure S3**).

**Table 4:** The details for the candidate genes of protein content and oil content.

Chromosome	Candidate gene	Region (Mb)	Annotation
*QPC3.1*	*Glyma.03G261000*	45465440–45469548	Pyruvate dehydrogenase (acetyl-transferring) kinase
*QPC6.1*	*Glyma.06G263800*	45124651–45126548	Acetyl-CoA carboxylase 1
*QPC7.1*	*Glyma.07G137400*	16298029–16299140	Acetyl-CoA carboxylase 2
*QPC10.1*	*Glyma.10G065000*	6209769–6215999	Pyruvate kinase, cytosolic isozyme-like
*QOC12.1*	*Glyma.12G014800*	1071368–1079413	Abscisic acid G-protein-coupled receptor
*QOC13.1*	*Glyma.13G119800*	23237418–23241011	Pyruvate dehydrogenase (acetyl-transferring) kinase
*QPC15.1*	*Glyma.15G049200*	3861401–3863151	SWEET (sugars will eventually be exported transporters) family
*QPC20.1*	*Glyma.20G189300*	42789586–42797140	Glycine max pyruvate kinase
*QOC8.1*	*Glyma.08G107800*	8296214–8307239	Bifunctional aspartate kinase/homoserine dehydrogenase (AK-HSDH)

*Glyma.03G261000* (chromosome 3: 45465440–45469548 bp) for *qPC3.1* (47.3–50.4 Mb) regulated pyruvate dehydrogenase (acetyl-transferring) kinase activity; *Glyma.10G065000* (chromosome 10: 6209769–6215999 bp) for *qPC10.1* (6.0–10.8 Mb) encoded pyruvate kinase; *Glyma.06G263800* (chromosome 6: 45124651–45126548 bp) for *qPC6.1* (45.1–47.4 Mb) encoded acetyl-CoA carboxylase 1; *Glyma.07G137400* (chromosome 7: 16298029–16299140 bp) for *qPC7.1* (3.9–8.4) encoded acetyl-CoA carboxylase 2; *Glyma.12G014800* (chromosome 12: 1071368–1079413 bp) for *qOC12.1* (2.1–7.4 Mb) is an abscisic acid G-protein-coupled receptor; *Glyma.13G119800* (chromosome 13: 23237418–23241011 bp) for *qOC13.1* (19.9–25.0 Mb) encoded pyruvate dehydrogenase (acetyl-transferring) kinase activity; *Glyma.20G189300* (chromosome 20: 42789586–42797140 bp) for *qPC20.1* (0.5–3.2 Mb) is a Glycine max pyruvate kinase, cytosolic isozyme; *Glyma.08G107800* (chromosome 8: 8296214–8307239 bp) for *qOC8.1* (8.3–8.4 Mb) encoded a bifunctional aspartate kinase/homoserine dehydrogenase (AK-HSDH); and *Glyma.15G049200* (chromosome 15: 3861401–3863151 bp) for *qPC15.1* (1.0–4.9 Mb) encoded a SWEET family.

## Discussion

The characterization of the subgroups for all 320 soybean accessions was largely consistent with geographical origins (Wang et al., 2018; Jeong et al., 2019; Kim et al., 2021; Torkamaneh et al., 2021). Most of the cultivars from China, Russia, and Ukraine belonged to subgroup I, the accessions mainly from the United States and Canada belonged to subgroup II, and subgroup III mainly comprised 62 varieties from Germany, France, and Czechia (**Figure 1**). To avoid spurious MTAs, an MLM model with PCA and kinship matrix was applied in this study (Zhu et al., 2008). LD decay influenced the precision of GWAS and was affected by allele frequency, population structure, and recombination rate (Liu et al., 2017). The LD decay of the whole genome was 450 kb, indicating that the marker density is sufficient for subsequent GWAS.

### Loci associated with protein and oil contents in previous studies

In the present study, *qPC2.2*, *qPC-7-1*, *qPC13.1*, *qPC15.1*, and *qPC20.1* related to the PC overlapped with previously reported loci, indicating the reliability of the results (Mao et al., 2013; Warrington et al., 2015; Zhou et al., 2015; Phansak et al., 2016; Zhang et al., 2021). Meta-QTLs were detected consistent with *qPC2.2*, *qPC-7-1*, and *qPC20.1* in various populations (Pathan et al., 2013; Warrington et al., 2015; Zhou et al., 2015; Kim et al., 2016; Phansak et al., 2016; Van and McHale, 2017). *qPC13.1* (24.3–28.6 Mb, explaining 5.2%–9.6% of phenotypic variation) on chromosome 13 was also reported (Mao et al., 2013; Zhang et al., 2021). Previous studies also identified the locus *qPC15.1* as an important QTL for PC in soybean (Warrington et al., 2015; Zhou et al., 2015; Phansak et al., 2016; Zhang et al., 2021).

QTL for OC was distributed on all chromosomes except chromosome 9. The loci *qOC2.2*, *qOC6.1*, *qOC6.2*, *qOC7.1*, *qOC8.1*, *qOC12.2*, *qOC19.2*, and *qOC20.2* overlapped with QTL related to oil and its compositional content in previous studies (Chung et al., 2003; Liang et al., 2010; Li et al., 2019; Zhang et al., 2019; Zhang et al., 2021). *qOC12.2* (9.7–10.1 Mb) overlapped with the loci for *qOC-12-1* (9.1–11.5 Mb), which was identified by Zhang et al. (2021) using 211 diverse soybean accessions genotyped with 355 K SoySNP array. *qOC8.1* (8.3–8.4 Mb) overlapped with *GqOil-8* (7.9–8.3 Mb), which was detected in several previous studies (Zhang et al., 2019; Zhang et al., 2021). *qOC6.1* (9.6–10.4 Mb) was also reported by Zhang et al. (2019) (8.3–9.6 Mb, explaining 7.3% of phenotypic variation) and (9.0–11.2 Mb, explaining 11.2% of phenotypic variation). *qOC2.2* (44.7–49.2 Mb) overlapped with the loci for *qOil2-1* (42.5–45.4 Mb), identified by Zhang et al. (2019) using 200 soybean accessions. *qOC6.2* on chromosome 6 (9.6–10.4 Mb) overlapped with the loci *qOC-6-1* (8.5–12.3 Mb), which has been reported by a series of studies with 6.3%–15.6% phenotypic variations (Diers et al., 1992; Hyten et al., 2004; Zhang et al., 2021). *qOC7.1* (11.7–15.6 Mb) overlapped with the loci (associated SNP marker *rs15774585*) from 185 soybean cultivars identified by Li et al. (2019) (10.2–13.0 Mb). *qOC8.1* (8.3–8.4 Mb) overlapped with previously reported QTL related to the PC with 5.2%–11.5% phenotypic variations (chromosome 8: 7.9–11.2 Mb) (Lu et al., 2013; Pathan et al., 2013). *qOC19.2* on chromosome 19 (43.9–46.8 Mb) was reported by Chapman et al. (2003) (44.4–47.2 Mb, explaining 7.9% of phenotypic variations) and Li et al. (2019) (41.5–44.6 Mb, explaining 6.9% of phenotypic variations), whereas *qOC20.2* (31.5–32.9 Mb, explaining 5.2%–11.5% of phenotypic variations) was reported previously by Bachlava et al. (2009) and Zhang et al. (2021). The remaining three QTLs related to the OC were novel. Among the loci identified for PC and OC, 13 loci mentioned above are probably the same as the QTL reported in previous studies, whereas the remaining loci are likely to be new. The stable loci validated by our studies and previous studies indicated that they are less affected by environmental factors.

### Potential candidate genes associated with protein and oil content

A total of nine candidate genes were identified for soybean PC and OC. *Glyma.15G049200* was identified in the LD block of *qPC15.1* and belonged to the SWEET family, which is involved in the transportation of carbohydrates and plays a vital role in transporting sucrose molecules across a membrane. The accumulation of storage substances promotes the development of seeds. Sucrose in seeds could be metabolized to produce protein precursors (Smith et al., 1989). *Glyma.08G107800* was located with the LD decay of *qPC8.1*. *Glyma.08G107800* encoded an AK-HSDH. AK-HSDH plays a vital role in the synthesis of amino acids for Lys, Ile, and Met (Kang et al., 2018). The aspartic acid family is the main component in the biosynthesis of other amino acids (Zhang et al., 2018; Zhang et al., 2021).

The oil and protein involved in carbohydrate transport in soybean seeds are complex, mainly including the carbon source competition and distribution process. The candidate gene of loci *qPC3.1* (*Glyma.03G261000*), *qPC10.1* (*Glyma.10G065000*), *qOC13.1* (*Glyma.13G119800*), and *qPC20.1* (*Glyma.20G189300*) encoded pyruvate dehydrogenase (acetyl-transferring) kinase, pyruvate kinase, pyruvate dehydrogenase (acetyl-transferring) kinase, and glycine max pyruvate kinase, respectively. The energy produced by photosynthesis is stored mainly in the form of proteins and lipids (Zhang et al., 2021). Pyruvate dehydrogenase is very important in carbon metabolism, the tricarboxylic acid (TCA) cycle, and glycolysis/gluconeogenesis (Zhang et al., 2019; Yao et al., 2020). Pyruvate dehydrogenase catalyzes the formation of pyruvate, which is the substrate of the Calvin cycle (Yao et al., 2020).

The candidate gene for *qPC6.1* (*Glyma.06G263800*) and *qPC7.1* (*Glyma.07G137400*) encoded acetyl-CoA carboxylase 1 and acetyl-CoA carboxylase 2, respectively. Acetyl-CoA carboxylase provides a carbon skeleton for the synthesis of fatty acids and plays a vital role in the glycolysis pathway (Alfonso, 2020). The metabolites are required for the formation of fatty acids by acetyl coenzyme (Alfonso, 2020; Megha et al., 2022). In addition, starch and sucrose produced by glycolysis can accelerate mitochondrial respiration and the TCA cycle, which is the most critical metabolic pathway for carbohydrate, protein, and fat oxidation (Allen et al., 2009; Liu et al., 2020). *Glyma.12G014800* is the candidate gene for *qOC12.1* and encoded an abscisic acid G-protein-coupled receptor. Abscisic acid affects the accumulation of assimilates (Manan and Zhao, 2020). G-protein promotes oil increase by regulating the abscisic acid signal transduction (Manan and Zhao, 2020).

### Implications of improving protein and oil content in soybean breeding

Conventional breeding has led to an increase in PC and OC (Li et al., 2020). However, selective breeding is time-consuming and costly (Qiu et al., 2013; Lin et al., 2022). The stable SNPs associated with PC and OC identified in this study, such as *qPC3.1*, *qPC5.2*, *qPC9.1*, *qPC10.1*, *qPC16.1*, *qOC1.2*, and *qOC11.1* for PC, and *qOC12.1*, *qOC15.1*, *qOC16.1*, and *qOC20.1* for OC, could be used for soybean MAS breeding, and pyramiding favorable alleles will improve PC and OC. Accessions with superior PC and OC alleles (such as R256, R188, R247, R200, R61, R238, R75, R31, R190, R156, R173, and R13 for OC, and R124, R70, R121, R74, R199, R127, R196, R144, R249, R248, R59, R54 and R134 for PC) could be used as parental lines for the molecular improvement of PC and OC in soybean.

## Conclusions

In this study, we have identified 23 and 29 loci for PC and OC in 320 soybean accessions, respectively. Of these, 15 and 24 loci are likely to be new. In addition, nine candidate genes involved in protein and oil biosynthesis/metabolism were identified, including lipid transport and metabolism, signal transduction, and plant development pathway. These significantly associated SNPs and varieties with favorable alleles could be used to accelerate the progress of breeding soybean with higher PC and OC.

## Data availability statement

The datasets presented in this study can be found in online repositories. The names of the repository/repositories and accession number(s) can be found in the article/**Supplementary Material**.

## Ethics statement

We declare that these experiments complied with the ethical standards in China.

## Author contributions

HJ performed the experiment and drafted the manuscript. HZ, XY, and XS analyzed the physiology data. YT, YW, and QG revised the manuscript. RZ and JZ designed the research. All authors contributed to the article and approved the submitted version.

## Supplementary material

Supplementary Figure 1 Manhattan plot for the protein content and oil content across all environments. PC: protein content; OC: oil content; E1, E2, E3 E4, E5, and E6 indicate 2017 Harbin, 2018 Harbin, 2019 Harbin, 2021 Harbin, 2018 Mudanjiang, and 2018 Qingan, respectively.

Supplementary Figure 2 Quantile–quantile (Q–Q) plot for protein content and oil content in all 320 soybean accessions analyzed by the mixed linear model (MLM) in Tassel v5.0. PC: protein content; OC: oil content; E1, E2, E3 E4, E5, and E6 indicate 2017 Harbin, 2018 Harbin, 2019 Harbin, 2021 Harbin, 2018 Mudanjiang, and 2018 Qingan, respectively.

Supplementary Figure 3 The expression of the candidate gene by qRT-PCR results.
