# Supplementary figures and images for: Genetic analysis of protein content and oil content in soybean by genome-wide association study
Source: Front Plant Sci. 2023 Jun 6;14:1182771. doi: 10.3389/fpls.2023.1182771 (PMC10281628; doi:10.3389/fpls.2023.1182771)

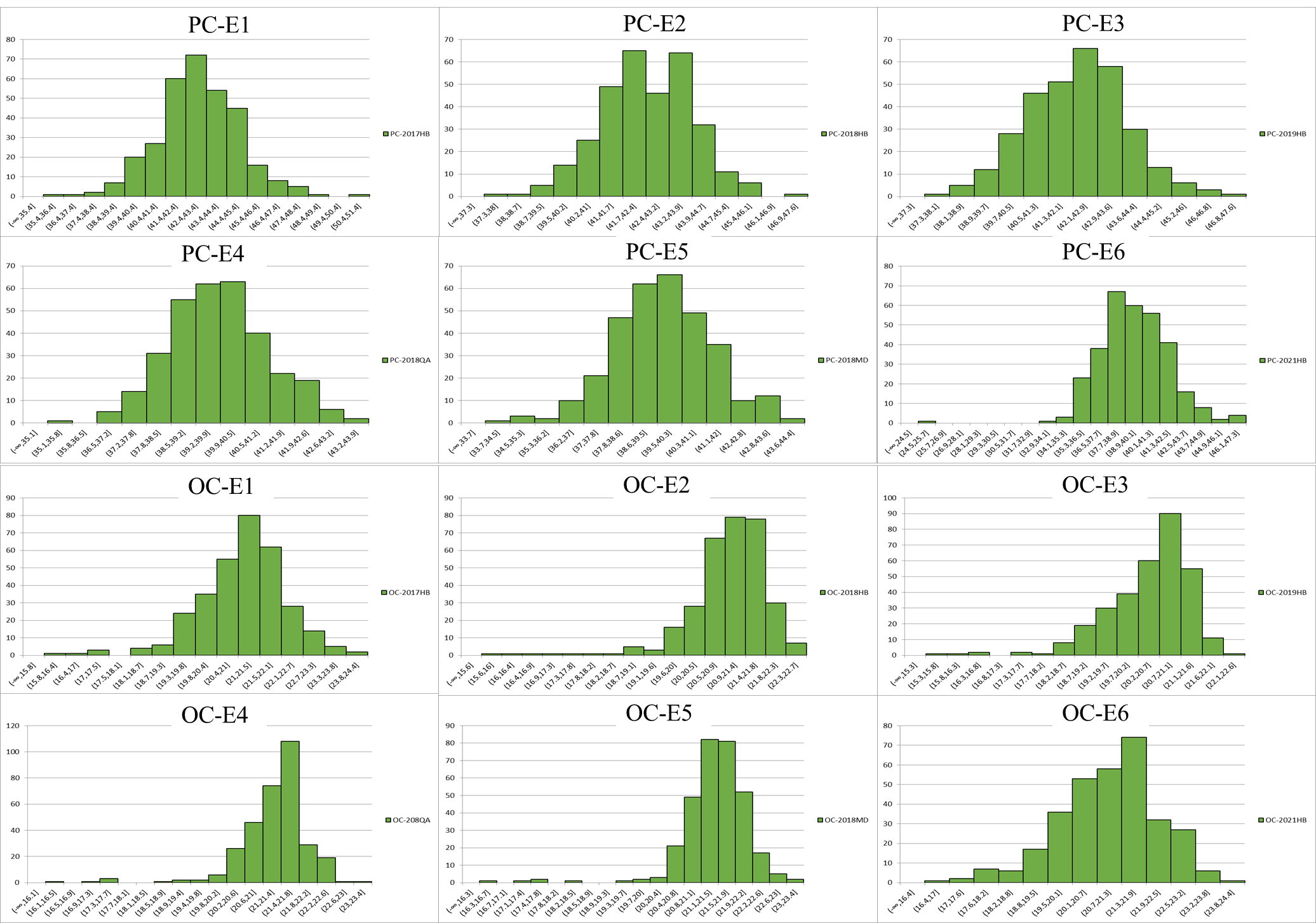

Supplement: Supplementary Figure 1 — Manhattan plot for the protein content and oil content across all environments. PC: protein content; OC: oil content; E1, E2, E3 E4, E5, and E6 indicate 2017 Harbin, 2018 Harbin, 2019 Harbin, 2021 Harbin, 2018 Mudanjiang, and 2018 Qingan, respectively. [file Image_1.tif]

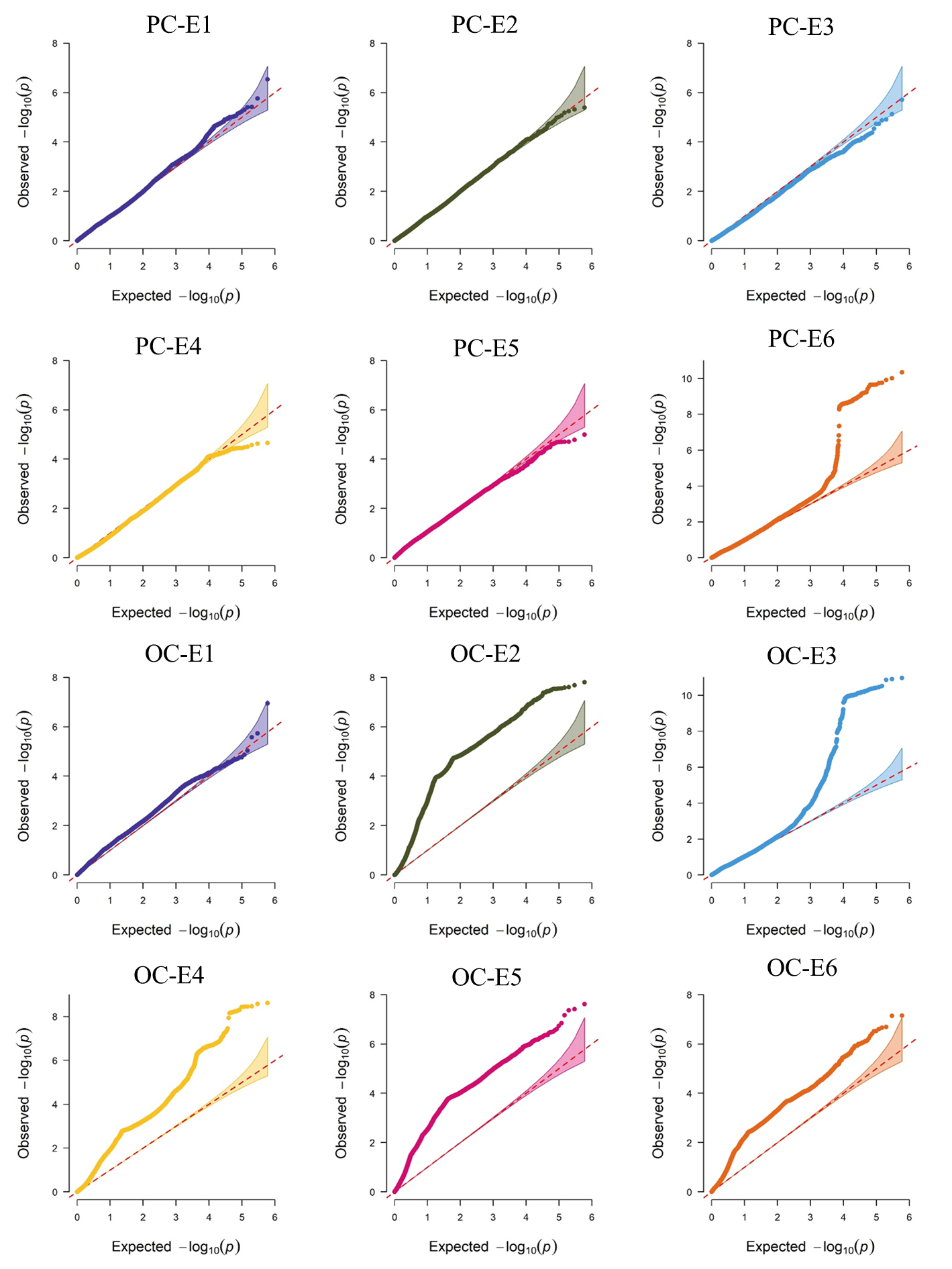

Supplement: Supplementary Figure 2 — Quantile–quantile (Q–Q) plot for protein content and oil content in all 320 soybean accessions analyzed by the mixed linear model (MLM) in Tassel v5.0. PC: protein content; OC: oil content; E1, E2, E3 E4, E5, and E6 indicate 2017 Harbin, 2018 Harbin, 2019 Harbin, 2021 Harbin, 2018 Mudanjiang, and 2018 Qingan, respectively. [file Image_2.tif]

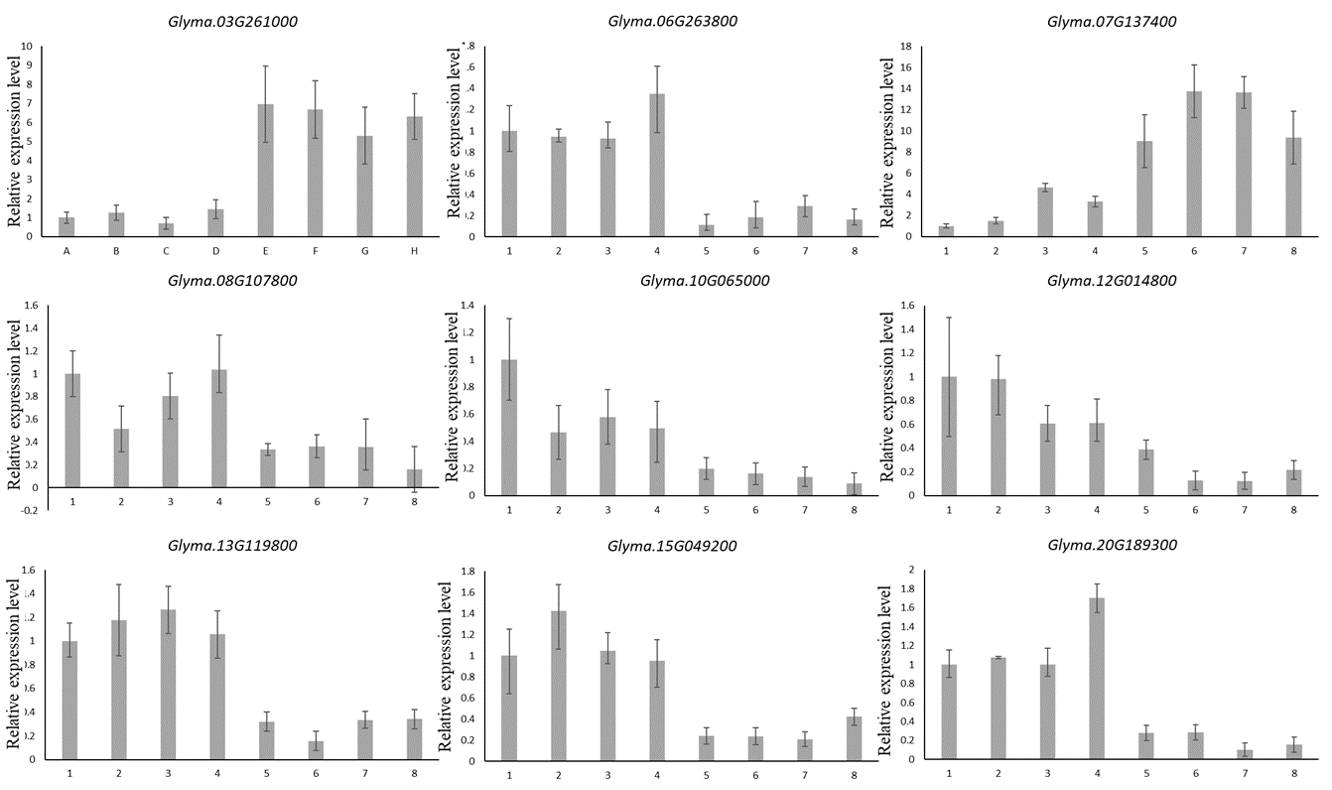

Supplement: Supplementary Figure 3 — The expression of the candidate gene by qRT-PCR results. [file Image_3.tif]
