# Supplementary material for: Genetic analysis of protein content and oil content in soybean by genome-wide association study
Source: Front Plant Sci. 2023 Jun 6;14:1182771. doi: 10.3389/fpls.2023.1182771 (PMC10281628; doi:10.3389/fpls.2023.1182771)
Supplement: Supplementary file 5 [file Table_2.docx]

**Table S2** qRT-PCR primer for the candidate genes

| Candidate gene | Primer-F | Primer-R |
| --- | --- | --- |
| *Glyma.03G261000* | ACGTTACAGACAAAATGCCCT | ATGTTGACCGTCAGGATTGC |
| *Glyma.06G263800* | GAGCTACGCGTTTGGATGGA | TGTCGGGACAGGAGGAAAGA |
| *Glyma.07G137400* | GAAGGTCAAGCAATGCAGGC | TGCAGTAGGAGGTCCCAGAA |
| *Glyma.08G107800* | TCACTTCGTTTCCGTGGAGC | AAGAAAGGGCGGCATTGAGA |
| *Glyma.10G065000* | ACAGCTACATTGGCTGGGTC | CGTCTTTAGCGTGCCGAGTA |
| *Glyma.12G014800* | AGTTTTTGCACATGGGATTGGT | TCAAGACAAACAAACGGCCA |
| *Glyma.13G119800* | TGGGACTGACGCATATCTCC | CGTCTGCATCGTTTTGGTGC |
| *Glyma.15G049200* | CGGTGAGAGTGAGAAGGAGC | GGCGGCCGTAAGCAATTAAA |
| *Glyma.20G189300* | TTGGGTGACCTGAAGCAGAC | CCACGAGCGAGGATGATACC |
