# Supplementary material for: Genetic analysis of protein content and oil content in soybean by genome-wide association study
Source: Front Plant Sci. 2023 Jun 6;14:1182771. doi: 10.3389/fpls.2023.1182771 (PMC10281628; doi:10.3389/fpls.2023.1182771)
Supplement: Supplementary file 6 [file Table_3.docx]

Table S3 The accessions with extreme protein content and oil content used for qRT-PCR in the 320 soybean accessions

| **Trait** | **Number** | **Extreme-higher** |
| --- | --- | --- |
| **Protein content** | 1 | C228 |
|  | 2 | C267 |
|  | 3 | C230 |
|  | 4 | C201 |
|  | 5 | C076 |
|  | 6 | C096 |
|  | 7 | C062 |
|  | 8 | C100 |
| **Oil content** | 1 | C236 |
|  | 2 | C147 |
|  | 3 | C059 |
|  | 4 | C200 |
|  | 5 | C076 |
|  | 6 | C096 |
|  | 7 | C062 |
|  | 8 | C100 |
